# Identify Bitter Peptides by Using Deep Representation Learning Features

**DOI:** 10.3390/ijms23147877

**Published:** 2022-07-17

**Authors:** Jici Jiang, Xinxu Lin, Yueqi Jiang, Liangzhen Jiang, Zhibin Lv

**Affiliations:** 1 College of Biomedical Engineering, Sichuan University, Chengdu 610065, China; jiangjici@foxmail.com; 2 College of Software Engineering, Sichuan University, Chengdu 610065, China; linxinxulx@foxmail.com; 3 West China School of Medicine, Sichuan University, Chengdu 610065, China; jiangyueqijyq@foxmail.com; 4 Key Laboratory of Coarse Cereal Processing, Ministry of Agriculture and Rural Affairs, College of Food and Biological Engineering, Chengdu University, Chengdu 610106, China; lzhjiang1987@sina.com

**Keywords:** bitter peptide, deep representation learning, light gradient boosting, feature selection

## Abstract

A bitter taste often identifies hazardous compounds and it is generally avoided by most animals and humans. Bitterness of hydrolyzed proteins is caused by the presence of bitter peptides. To improve palatability, bitter peptides need to be identified experimentally in a time-consuming and expensive process, before they can be removed or degraded. Here, we report the development of a machine learning prediction method, iBitter-DRLF, which is based on a deep learning pre-trained neural network feature extraction method. It uses three sequence embedding techniques, soft symmetric alignment (SSA), unified representation (UniRep), and bidirectional long short-term memory (BiLSTM). These were initially combined into various machine learning algorithms to build several models. After optimization, the combined features of UniRep and BiLSTM were finally selected, and the model was built in combination with a light gradient boosting machine (LGBM). The results showed that the use of deep representation learning greatly improves the ability of the model to identify bitter peptides, achieving accurate prediction based on peptide sequence data alone. By helping to identify bitter peptides, iBitter-DRLF can help research into improving the palatability of peptide therapeutics and dietary supplements in the future. A webserver is available, too.

## 1. Introduction

Humans and most animals instinctively dislike bitter substances, as the taste often identifies toxic compounds. However, some beneficial nutrients, such as soy products, endive, and other Asteraceae vegetables, as well as certain therapeutic peptides, are often bitter. Proteins can be enzymatically digested into shorter polypeptides that have certain beneficial biological activities. Studies have shown that hydrolyzed polypeptides have good nutritional properties and can be easily absorbed and utilized. However, hydrolysis often produces peptides with varying degrees of bitterness that can be detected even at very low concentrations [1]. The bitter taste of protein hydrolysates is caused by the presence of peptides containing hydrophobic amino acids. Most of these peptides are typically composed of no more than eight amino acids and few contain more than ten. However, bitter peptides containing up to 39 amino acids have been described [2]. The bitter taste of protein hydrolysates is the result of a variety of factors. Hydrophobic amino acids within the polypeptide tend to become exposed, stimulating the taste buds, causing the bitterness. Generally, the more hydrophobic amino acids are exposed, the stronger the bitter taste. In addition, the length, overall hydrophobicity, sequence, and amino composition of a polypeptide chain also have a significant impact on bitterness [3].

Identifying bitter peptides using conventional laboratory approaches is expensive and time-consuming. With the availability of a large number of peptide sequence databases in the post-genomic era [4], the development of automated computational models to identify novel bitter peptides has far-reaching practical implications. The success of machine learning-based peptide prediction methods led to an increased interest in bioinformatics [5,6,7,8]. A number of computational methods based on quantitative structure-activity relationship modeling (QSAR) and machine learning have been developed to predict the bitterness of polypeptides [9,10,11,12,13,14]. For example, BitterX [15], BitterPredict [16], iBitter-SCM [17], and iBitter-Fuse [18] using traditional sequence features were proposed to identify bitter peptides and showed increasing performance. Now the methods for identifying bitter peptides sequences are focused on feature engineering. The more meaningful the representation features for sequence are, the better the accuracy of the models will be. In 2021, BERT4Bitter [19] was proposed, which used natural language processing (NLP) heuristic signature coding methods to represent peptide sequences as feature descriptors, and it displayed better accuracy. While in 2022, He et al. [20] proposed mutual information-based meta learning (MIMML) to discover the best feature combination for bitter peptides and attained an independent accuracy of 93.8%. Although great progress has been made in this field, there is still much room for improvement in the performance of machine learning-based bitter peptide identification models using sequences only.

Deep learning refers to an algorithm in machine learning that does not require pre-processing or prior characterization of data, allowing the transformation of raw protein sequences into a form that machine learning can effectively utilize [21]. Inspired by deep learning application in the NLP area, sequence-based deep representation learning of proteins and peptides has emerged as an efficient and accurate method for predicting the characteristics of proteins [22,23,24], such as UniRep [25] and TAPE [26]. The models with deep representation learning features have been applied for identifying anticancer peptides [23], protein subcellular location [27], and post-translational modifications of proteins [28], showing significant improvement of models using traditional sequence features.

**Figure 1:**
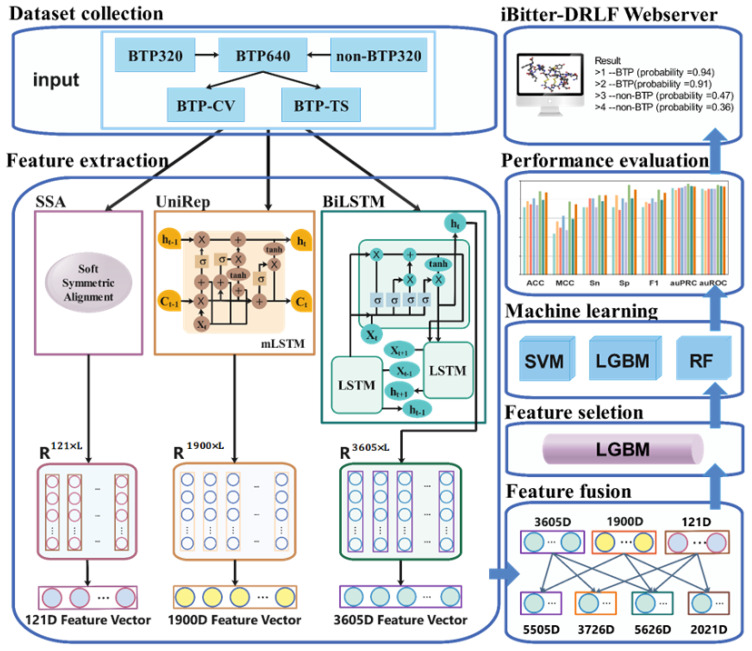
Overview of model development. The pre-trained SSA sequence embedding model, UniRep sequence embedding model, and BiLSTM sequence embedding model were used to embed peptide sequences into eigenvectors. Peptide sequences were converted into 121-dimensional (D) SSA eigenvectors, 1900-dimensional UniRep eigenvectors, and 3605-dimensional (D) BiLSTM eigenvectors. Features were combined and fused to derive the following fusion features: SSA-UniRep (2021D), SSA-BiLSTM (3726D), UniRep-BiLSTM (5505D), and SSA-UniRep-BiLSTM (5626D). These fusion features were used as inputs to the SVM, RF, and LGBM predictor algorithms. The model was optimized by feature selection using the LGBM method. Selected feature sets were subjected to another round of analysis using three algorithms and various hyperparameters. Through 10-fold cross-validation and comparison of independent tests results, the optimized final model was developed. Here, the example like SSA-BiLSTM means two kind of features are combined.

Here, we used two protein/peptide sequence-based deep representation learning feature to develop a machine learning based model named iBitter-DRLF for bitter peptides identification. It achieved impressive 10-fold cross-validation results, and independent test accuracy compared favorably with existing methods and traditional, non-deep representation learning methods. Furthermore, iBitter-DRLF could be used more generally and showed superior discrimination in detecting bitter peptides. In addition, based on the results of independent tests, iBitter-DRLF (ACC = 0.944, MCC = 0.889, Sp = 0.977, F1 = 0.952, auPRC = 0.984, auROC = 0.977) outperformed the most advanced predictors currently available. Apart from the feature analysis methods, unified manifold approximation and projection dimensionality reduction (UMAP) was also used to explore the effects of different feature analysis approaches and different depth characterization learning features on model performance. The user-friendly webserver is available at https://www.aibiochem.net/servers/iBitter-DRLF/ (accessed on 1 May 2022). A diagram describing the steps taken during the development of iBitter-DRLF is shown in Figure 1.

## 2. Result and Discussion

### 2.1. Results of Preliminary Optimization

To explore embedded features that are useful in identifying bitter peptides, we first used three deep representation learning feature extraction methods, soft symmetric alignment (SSA), unified representation (UniRep), and bidirectional long short-term memory (BiLSTM). To generate each of these, we used three distinct machine learning methods, SVM, LGBM, and RF, to develop models and carry out their initial optimization. Table 1 shows the results of 10-fold cross-validation and independent tests for the three models developed based on the above assumptions. The values in the table represent model performance measures after the optimization of model parameters. The best values achieved for individual features are shown in bold and are underlined.

As shown in Table 1, the 10-fold cross-validation results of the UniRep feature vector, developed using SVM, performed the best of all tested feature/model combinations (accuracy (ACC) = 0.865, Matthews correlation coefficient (MCC) = 0.730, sensitivity (Sn) = 0.867, specificity (Sp) = 0.863, F1 = 0.865, area under the PRC curve (auPRC) = 0.937, and area under the ROC curve (auROC) = 0.931). The ACC of this feature vector exceeded other options by 1.17–9.91%. MCC was better by 2.67–26.69%, Sn by 0.46–6.25%, Sp by 1.77–14.46%, F1 by 0.98–9.08%, and auROC by 0.54–7.63%. Regarding its performance in independent tests (ACC = 0.867, MCC = 0.735, Sn = 0.844, Sp = 0.891, F1 = 0.864, auPRC = 0.952, auROC = 0.948), ACC was 1.85% lower, MCC was reduced by 4.22%, Sn was reduced by 7.35%, F1 was reduced by 2.49%, and auPRC by 0.47% compared to the BiLSTM feature vector developed based on SVM. It can be concluded that, for the identification of bitter peptides, UniRep features were superior to BiLSTM features.

### 2.2. The Effects of Feature Fusion on the Automatic Identification of Bitter Peptides

**Figure 2:**
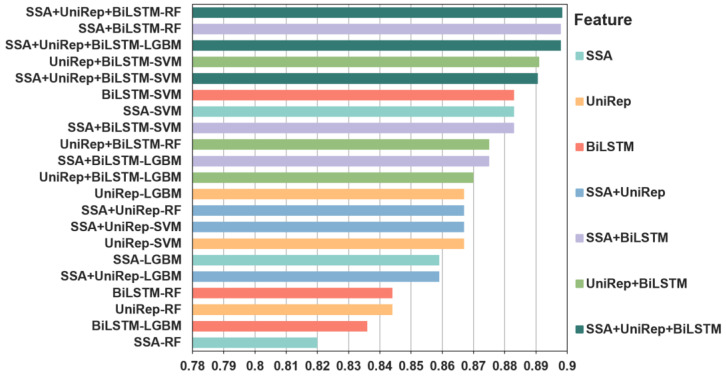
The accuracy of independent tests for predicting individual and fused features of bitter peptides. Three individual features, SSA, UniRep, and BiLSTM, three double fusion features, SSA + UniRep, SSA + BiLSTM, and UniRep + BiLSTM, and a triple fused feature, SSA + UniRep + BiLSTM, were tested with three distinct machine learning algorithms. The same color is used to signify the same features. The accuracy of the individual or fused feature/machine learning algorithm combinations is ranked from highest to lowest. The accuracy of one of the triple fusion features and four fused double features outperformed the best performing individual feature. In contrast, the four least accurate predictors were individual feature models, indicating the superiority of fused features. The accuracy of the SSA + UniRep + BiLSTM-LGBM) combination was 0.898. The best performing SSA-SVM and BiLSTM-SVM individual features had an accuracy of 0.883. In summary, combining different feature information sets helped to improve the predictive performance of the model. Please note SSA-SVM means that the SVM model with SSA feature vectors as input while and SSA + UniRep + BiLSTM-LGBM means that the LGMB model with the combination of SSA, UniRep and BiLSTM features as input. Other similar labels have similar meanings.

At this stage of the development work, we evaluated the use of pairwise combinations of features to generate fusion features. Features were combined in all possible pairings, namely, SSA + UniRep, SSA + BiLSTM, and UniRep + BiLSTM. In addition, SSA, UniRep, and BiLSTM were combined to obtain a triple fusion feature, SSA + UniRep + BiLSTM. These fusion feature combinations were used as input into SVM, LGBM, and RF algorithms to train predictive models and to optimize model performance (Figure 2). Table 2 shows the 10-fold cross-validation and independent test results for all the developed models. The values in this table represent the results after optimizing model parameters. Again, the best performance metrics values are shown in bold and are underlined.

Comparing Table 1 (see Section 3.1) and Table 2, it is immediately apparent that the optimal performance values of the models using fusion features are better than the best values obtained with non-combined features. As an example, the performance of the combination of the 121-dimensional SSA feature and the 5505-dimensional BiLSTM, SSA + BiLSTM, developed using RF, showed an ACC value of 0.898, while the ACC of the SSA feature alone was 0.820, representing a 9.51% better performance of the fusion feature.

### 2.3. The Effects of Feature Selection on the Automatic Identification of Bitter Peptides

**Figure 3:**
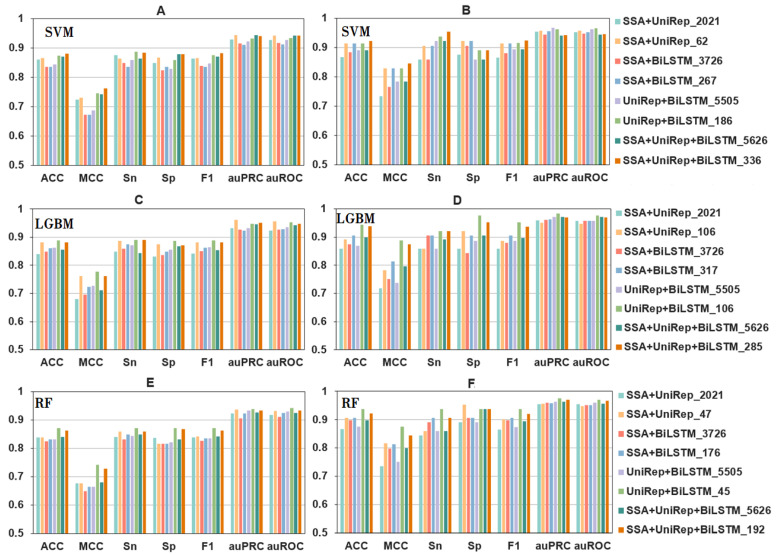
The performance metrics of fusion features using a range of selected features and different algorithms. Panels (**A**,**C**,**E**) show 10-fold cross-validation results, and panels (**B**,**D**,**F**) are independent test results. Please note that the same colors represent different selected features in the different panels. Refer to the codes directly underneath a panel for the appropriately selected feature/model combination codes.

As described in previous section, fused feature encoding was clearly superior to non-fused feature encoding. The sequence vector used in the training set had 512 dimensions, while the feature vectors based on the combined fusion feature scheme had 2021, 3726, 5505, and 5626 dimensions. This high number of dimensions increases the risk of redundancy and the overfitting of feature information. To resolve this problem, we used the LGBM algorithm for feature selection, while using an incremental feature strategy and a hyperparametric mesh search method. For the latter, we selected the scikit-learn GridSearchCV module to perform the hyperparameter search for each model. The performance metrics of each individual, double, and triple fused feature developed using all three machine learning models (SVM, LGBM, RF) are summarized in Table 3, while a visual representation of the outcomes is shown in Figure 3.

The outcome measures shown in Figure 3 and Table 3 clearly indicate that the selected fusion feature sets performed significantly better than unselected fusion features. It is apparent from these results that the overall performance of the 106D UniRep + BiLSTM feature vector was better than outcomes achieved with any other feature vector. The results of 10-fold cross-validation (ACC = 0.899, MCC = 0.777, Sn = 0.891, Sp = 0.887, F1 = 0.889, auPRC = 0.947, auROC = 0.952) outperformed the ACC of other feature sets by 0.91–5.83%. MCC was better by 1.97–14.26%, Sn by 0.45–5.57%, Sp by 1.37–6.61%, and F1 by 0.79–5.70%. The independent test results for the 106D UniRep + BiLSTM fusion feature (ACC = 0.944, MCC = 0.889, Sn = 0.922, Sp = 0.977, F1 = 0.952, auPRC = 0.984, auROC = 0.977) were also better by 0.64–9.90% (ACC), 1.60–23.64% (MCC), 1.77–7.33% (Sp), 2.52–15.76% (Sn), 1.65–10.78% (F1), 1.23–3.42% (auPRC), and 0.62–3.17% (auROC).

These results clearly show that selecting feature descriptors is an effective way of resolving problems with information redundancy and was beneficial in optimizing the prediction performance of the bitter peptide prediction model.

### 2.4. The Effect of Machine Learning Model Parameter Optimization on the Automated Identification of Bitter Peptides

It is apparent from Table 3 that the overall performance of the SSA + UniRep_106 feature set was superior to all other options. There were only two isolated exceptions to this statement. The ten-fold cross-validation result developed based on LGBM with an auROC = 0.957 and the Sn of SSA + UniRep + BiLSTM_336 feature developed based on SVM (Sn = 0.953) were marginally better. Although these measures are 0.52% and 3.36% higher than those achieved using the UniRep + BiLSTM_106 feature, we believe that the UniRep + BiLSTM_106 feature developed using LGBM provided the best overall performance. Therefore, the UniRep + BiLSTM feature set was selected for final development, using three different machine learning methods to build models.

**Figure 4:**
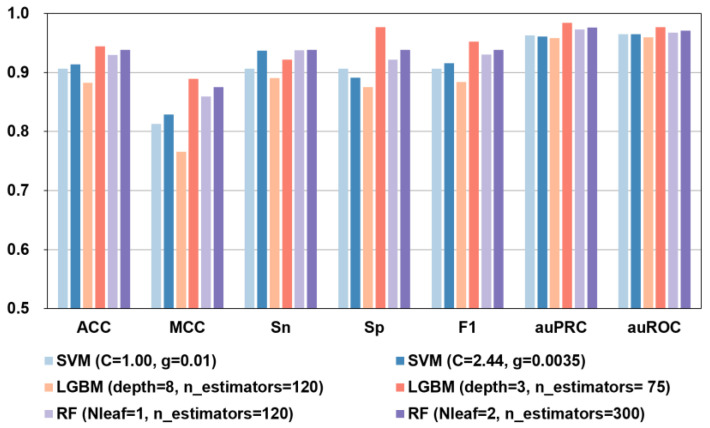
Performance metrics of the UniRep + BiLSTM features analyzed by different models using default parameters (light bars) or hyperparameters (dark bars). Results using selected hyperparameters invariably matched or outperformed default ones.

We utilized the scikit-learn GridSearchCV module to perform a hyperparameter search on each model, recording the corresponding optimal hyperparameters for each, and comparing them with default parameters. The observed values are shown in Figure 4 and in Supplemental Table S1.

As shown in Figure 4 and Supplemental Figure S1, when the UnRep + BiLSTM feature prediction was run using different algorithms and hyperparameters, the best performance was seen with the RF (Nleaf = 2, n_estimators = 300) model and the LGBM (depth = 3, n_estimators = 75) model. Although in the independent tests the Sn = 0.938 of the RF model was marginally better (Sn was 1.73% higher), in every other respect, including ACC, MCC, Sp, F1, auPRC, and auROC, the LGBM-based model showed clearly superior performance in both independent testing and 10-fold cross-validation.

Based on the analysis above, we selected the first 106D features of UniRep + BiLSTM to build the iBitter-DRLF predictor based on the LGBM model and selected the parameter depth = 3 and n_estimator = 75 values for further use. Although in 10-fold cross-validation the auPRC = 0.955 of LGBM model was marginally better (auPRC was 0.84% higher), in every other respect, the LGBM (depth = 3, n_estimators = 75) model showed clearly superior performance in both independent testing and 10-fold cross-validation. The 10-fold cross-validation results of this final prediction model were ACC = 0.889, MCC = 0.777, Sn = 0.891, Sp = 0.887, F1 = 0.889, auPRC = 0.947, and auROC = 0.952. The corresponding independent test results were ACC = 0.944, MCC = 0.889, Sn = 0.922, Sp = 0.977, F1 = 0.952, auPRC = 0.984, and auROC = 0.977.

### 2.5. Comparison with Existing Methods

We compared the predictive performance of iBitter-DRLF with existing methods, including iBitter-Fuse [18], MIMML [20], iBitter-SCM [17], and BERT4Bitter [19] to assess the effectiveness and utility of our method against its competitors. Independent test results for iBitter-DRLF and the existing methods are compared in Table 4. These results clearly demonstrate that iBitter-DRLF has significantly better ACC, MCC, Sp, and auROC, than existing methods. ACC outperformed other methods by 0.64–11.85%. MCC was 1.60–29.22% better, Sp was 4.16–15.76% higher, while auROC values were up by 1.35–8.08%. These comparisons show that iBitter-DRLF is more reliable and stable than existing algorithms in predicting the bitterness of peptides.

### 2.6. Feature Visualization of the Picric Peptide Automatic Recognition Effect

**Figure 5:**
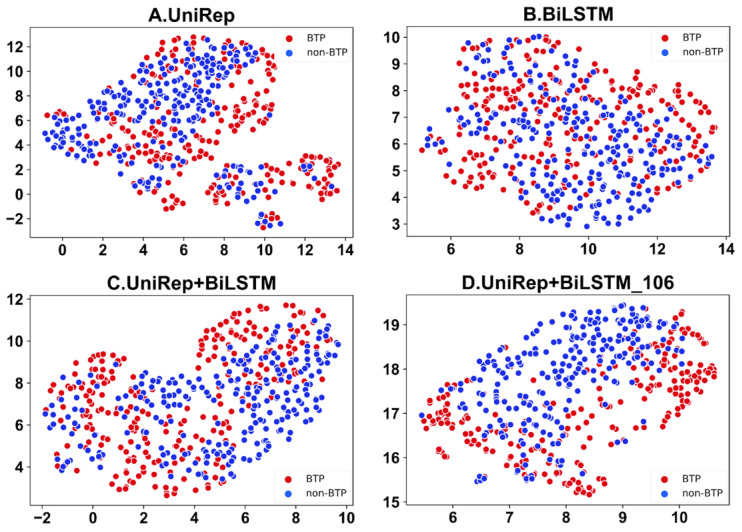
UMAP was used to visualize the dimension-reduced features of fused features: (**A**) is the UniRep feature, (**B**) is the BiLSTM feature, (**C**) is the BiLSTM + UniRep fusion, and (**D**) represents data using the first 106 features selected from the BiLSTM_UniRep fusion feature set.

Feature visualizations can communicate key data and features through graphics and colors to enable better insight into complex datasets. UMAP is a consistent manifold approximation and projection for the reduction of dimensionality. This algorithm is also suitable for the visual analysis of peptide characteristics. Feature visualization analysis of the automatic recognition of bitter peptides carried out by the UMAP algorithm preserved the characteristics of the original data well while greatly reducing the dimensions of characteristics. Through UMAP feature visualization, differences in feature representation can be clearly shown. Furthermore, the reasons for performance improvements of the model after feature optimization can be explained. The visualization of dimension-reduced features achieved using UMAP is shown in Figure 5. Apparently as compared to Figure 5 A–C, the first 106 features of the UniRep and BiLSTM fusion function, shown in Figure 5D, can better discriminate bitter peptides from non-bitter ones.

### 2.7. iBitter-DRLF Webserver

To facilitate the widespread use of our algorithm, we developed an iBitter-DRLF webserver that is freely available online at https://www.aibiochem.net/servers/iBitter-DRLF/ (accessed on 1 May 2022) to other investigators for the prediction of bitter peptides. The webserver is easy to use. Just pasting the peptide sequences into the text box, clicking the run button and waiting for a few minutes, the results will be displayed in the web pages. Please see the webserver interface at the website or in Supplementary Figures S2–S4.

## 3. Materials and Methods

### 3.1. Benchmark Dataset

The updated benchmark dataset from iBitter-SCM [17] is utilized here for modeling and to make future comparisons easier. Both peptides constructed as non-bitter using the BIOPEP database [29] and those previously experimentally confirmed to be bitter were included in the datasets used in this study. There are 320 bitter peptides and 320 non-bitter peptides in the BTP640 benchmark dataset. The dataset was randomly split into a training subset known as BTP-CV and an independent subset of test peptides known as BTP-TS in order to prevent overfitting the prediction model. The BTV-CV and BTS-TS groups had peptide ratios of 4:1. As a result, while the BTP-TS dataset contains 64 peptides in each category, the BTP-CV dataset contains 256 bitter peptides and 256 non-bitter peptides. Users can obtain both datasets from https://www.aibiochem.net/servers/iBitter-DRLF/ (accessed on 1 May 2022).

### 3.2. Feature Extraction

To explore the effects of different features on bitter peptide recognition, we used three deep representation learning feature extraction methods, namely, SSA [30], UniRep [25], and BiLSTM [31]. Models were trained on an alternate dataset for the identification of bitter peptides. Different feature encoding schemes were compared to build more comprehensive predictive models.

#### 3.2.1. Pre-Trained SSA Embedding Model

SSA defines a novel measure of similarity between sequences of arbitrary lengths embedded in vectors. First, a peptide sequence was used as input to a pre-trained model and encoded through a three-tier stacked BiLSTM encoder output. The final embedding matrix of each peptide sequence was obtained through a linear layer, $R^{L\times 121}$, where L is the length of the peptide. Such a model trained and optimized using SSA is referred to as an SSA-embedded model.

Suppose there are two embedded matrix of $R^{L\times 121}$ named $F_{1}$ and $F_{2}$ for two different peptide sequences of differing lengths, called $L_{1}$ and $L_{2}$.

(1) $$F_{1}=\left[x_{1},x_{2},\cdots ,x_{L1}\right],$$

where $x_{i}$ is a vector of 121D.

(2) $$F_{2}=\left[y_{1},y_{2},\cdots ,y_{L2}\right]$$

where $y_{i}$ is also a vector of 121D.

To calculate the similarity between two amino acid sequences represented as $F_{1}$ and $F_{2}$ separately, a soft symmetry alignment mechanism was developed, in which the similarity between the two sequences was calculated based on their embedded vector as follows:

(3) $$\hat{s}=-\frac{1}{A}\overset{L1}{\underset{i=1}{\sum }}\overset{L2}{\underset{j=1}{\sum }}a_{\mathrm{ij}}{∥x_{i}-y_{j}∥}_{1}$$

$a_{\mathrm{ij}}$ is determined by the following Formulas (4)–(7).

(4) $$\varphi_{\mathrm{ij}}=\frac{\exp\left(-{∥x_{k}-y_{j}∥}_{1}\right)}{\sum_{k=1}^{L1}\exp\left(-{∥x_{k}-y_{j}∥}_{1}\right)}$$

(5) $$\omega_{\mathrm{ij}}=\frac{\exp\left(-{∥x_{i}-y_{k}∥}_{1}\right)}{\sum_{k=1}^{L2}\exp\left(-{∥x_{i}-y_{k}∥}_{1}\right)}$$

(6) $$a_{\mathrm{ij}}=\omega_{\mathrm{ij}}+\varphi_{\mathrm{ij}}-\omega_{\mathrm{ij}}\varphi_{\mathrm{ij}}$$

(7) $$A=\overset{L1}{\underset{i=1}{\sum }}\overset{L2}{\underset{j=1}{\sum }}a_{\mathrm{ij}}$$

These parameters are backfitted with the parameters of the sequence encoder by a fully differentiated SSA. The trained model converts the peptide sequence into an embedding matrix, $R^{L\times 121}$, and a 121D SSA feature vector is generated by averaging pooling operations.

#### 3.2.2. Pre-Trained UniRep Embedding Model

The UniRep model was trained on 24 million UniRef50 primary amino acid sequences. The model performs next amino acid prediction by minimizing cross-entropy losses, thus learning how to represent proteins internally in the process. Using the trained model, a single fixed-length vector representation of the input sequence was generated by mLSTM (hidden state). The output vector representation was then trained into the best machine learning model. This characterizes the input sequence and enables supervised learning during different bioinformatics tasks.

First, the sequence with L amino acid residues was embedded into a matrix using a single thermal code, $R^{L\times 10}$. The matrix was then fed into the mLSTM encoder to obtain a hidden state output of $R^{1900\times L}$ as an embedding matrix. Finally, by an averaging pooling operation, the UniRep feature vector of 1900D was derived.

The calculation of the mLSTM encoder involves the following Equations (8)–(14).

(8) $$m_{t}=\left(X_{t}W_{\mathrm{xm}}\right)⊗\left(h_{t-1}W_{\mathrm{hm}}\right)$$

(9) $${\hat{h}}_{t}=\tan h\left(X_{t}W_{\mathrm{xh}}+m_{t}W_{\mathrm{mh}}\right)$$

(10) $$f_{t}=\sigma \left(X_{t}W_{\mathrm{xf}}+m_{t}W_{\mathrm{mf}}\right)$$

(11) $$i_{t}=\sigma \left(X_{t}W_{\mathrm{xi}}+m_{t}W_{\mathrm{mi}}\right)$$

(12) $$o_{t}=\sigma \left(X_{t}W_{\mathrm{xo}}+m_{t}W_{\mathrm{mo}}\right)$$

(13) $$C_{t}=f_{t}⊗C_{t-1}+i_{t}⊗{\hat{h}}_{t}$$

(14) $$h_{t}=o_{t}⊗\tan h(C_{t})$$

In these equations, ⊗ represents element-by-element multiplication, $h_{t-1}$ represents the previous hidden state, $X_{t}$ is the current input, and $m_{t}$ is the current intermediate multiplication state. ${\hat{h}}_{t}$ represents the input before the hidden state, $f_{t}$ is the forgotten gate, $i_{t}$ is the input gate, and $o_{t}$ is the output gate. $C_{t-1}$ is the previous unit state, $C_{t}$ is the current unit state, and $h_{t}$ is the hide state for output. σ is a sigmoid function, while tanh it is a tangent function.

#### 3.2.3. Pre-Trained BiLSTM Embedding Model

BiLSTM is a combination of a forward LSTM and a backward LSTM that captures bidirectional sequence features better than either LSTM model. LSTM obtains the ability to calculate by forgetting and memorizing information. This propagates information that is useful for subsequent computation moments to pass through while discarding useless information and outputting hidden states at each time point. The forgetting memory and output are controlled by the forgetting gate, memory gate, and output gate. These gates are calculated from the hidden state of the previous moment and the current input.

The calculation of BiLSTM involves the following Formulas (15)–(20).

(15) $$f_{t}=\sigma \left(W_{f}\cdot \left[h_{t-1},X_{t}\right]+b_{f}\right)$$

(16) $$i_{t}=\sigma \left(W_{i}\cdot \left[h_{t-1},X_{t}\right]+b_{i}\right)$$

(17) $${\tilde{C}}_{t}=\tan h\left(W_{C}\cdot \left[h_{t-1},X_{t}\right]+b_{C}\right)$$

(18) $$o_{t}=\sigma \left(W_{o}\cdot \left[h_{t-1},X_{t}\right]+b_{o}\right)$$

(19) $$C_{t}=f_{t}*C_{t-1}+i_{t}*{\tilde{C}}_{t}$$

(20) $$h_{t}=o_{t}*\tan h(C_{t})$$

Here $X_{t}$ is the current input, $h_{t-1}$ represents the previous hidden state, ${\tilde{C}}_{t}$ is the current cell state, $f_{t}$ is the forgotten gate, $i_{t}$ is the input gate, $o_{t}$ is the output gate, $C_{t-1}$ is the previous cell state, and $h_{t}$ is the output hidden state. Again, σ is a sigmoid function, while tanh is a tangent function.

#### 3.2.4. Feature Fusion

To establish the best feature combination, first we combined the SSA eigenvector of 121D with the UniRep eigenvector of 1900D, obtaining the SSA + UniRep fusion feature vector, 2021D. Second, the SSA eigenvector of 121D was combined with the BiLSTM eigenvector of 3605D to obtain the 3726D SSA + BiLSTM fusion eigenvector. Third, the 1900D UnIrep eigenvector was combined with the 3605D BiLSTM eigenvector giving the 5505D UniRep + BiLSTM fusion feature vector. Finally, the 121D SSA eigenvector, the 1900D UniRep eigenvector, and the 3605D BiLSTM eigenvector were combined to obtain the 5626D SSA + UniRep + BiLSTM fusion eigenvector.

### 3.3. Feature Selection Method

LGBM is a gradient boosting framework that abandons the level-wise decision tree growth strategy used by most gradient boosting tools in favor of a leaf-wise algorithm with depth restrictions. In this project, LGBM was utilized to identify the optimal feature space and sort features based on their importance values. Data and data labels were entered into the LGBM model to fit the model before using the built-in functions of LGBM to obtain the importance value for each feature. Features were ranked from ‘largest’ to ‘smallest’ based on feature importance values and those with an importance value greater than the critical value (the average feature importance values) were selected.

### 3.4. Machine Learning Methods

This work utilized three widely used high performance machine learning models, SVM [32,33], RF [34,35], and LGBM [23,36].

SVM is a typical machine learning (ML) algorithm for dealing with binary classification problems in bioinformatics. We chose gamma and C in the range: a row vector of 30 elements logarithmically divided from 10^−4^ to 10^4^. ‘rbf’ is the default kernel.

RF is a bagging-based algorithm that not only randomly selects samples, but also randomly selects features during the node splitting process. We selected the range of n estimators as (25, 550) and the range of Nleaf as (2, 12).

LGBM is a gradient boosting framework that uses tree-based learning algorithms. We selected the range of n estimators as (25, 750) and the range of max_depth as (1, 12).

### 3.5. Evaluation Metrics and Methods

We utilized the following five widely used measures [37,38] to evaluate the performance of specific models, calculated as follows: (21)–(25).

(21) $$\mathrm{ACC}=\frac{\mathrm{TP}+\mathrm{TN}}{\left(\mathrm{TP}+\mathrm{TN}+\mathrm{FP}+\mathrm{FN}\right)}$$

(22) $$\mathrm{MCC}=\frac{\mathrm{TP}\times \mathrm{TN}-\mathrm{FP}\times \mathrm{FN}}{\sqrt{\left(\mathrm{TP}+\mathrm{FP}\right)\left(\mathrm{TP}+\mathrm{FN}\right)\left(\mathrm{TN}+\mathrm{FP}\right)\left(\mathrm{TN}+\mathrm{FN}\right)}}$$

(23) $$\mathrm{Sn}=\frac{\mathrm{TP}}{\left(\mathrm{TP}+\mathrm{FN}\right)}$$

(24) $$\mathrm{Sp}=\frac{\mathrm{TN}}{\left(\mathrm{TN}+\mathrm{FP}\right)}$$

(25) $$F1=\frac{2\times \mathrm{TP}}{\left(2\times \mathrm{TP}+\mathrm{FN}+\mathrm{FP}\right)}$$

In these equations, TP represents the number of bitter peptides correctly predicted to be bitter, TN is the number of non-bitter peptides correctly predicted as non-bitter. FP represents the number of non-bitter peptides erroneously predicted as bitter, while FN is the number of bitter peptides falsely predicted as non-bitter. Using the auROC, the proposed models could be compared with each other and with previously described models. The precision-recall curve is the line connecting the points of precision and recall. Using the auPRC to represent the area enclosed by the precision-recall curve and the x-axis. The area under the ROC curve was also used to evaluate predicted performance, where AUC values ranging from 0.5 to 1 represent stochastic and perfect models, respectively.

K-fold cross-validation and independent testing are widely used to evaluate machine learning models. K-fold cross-validation divides the raw data into K-folds. One is used for the validation of each data subset, while the remaining K − 1 subset is used as the training set. In the validation set, K models are evaluated separately, and the final values of the model measures are averaged to obtain cross-validated values. During the work presented here, we used the 10-fold (K = 10) cross-validation method. For stand-alone testing, a completely different dataset from the training set was used, where all the samples were new to the trained model.

## 4. Conclusions

Here we describe the development of a new computational model called iBitter-DRLF that can accurately identify bitter peptides based on sequence data alone. It uses a deep representation learning feature embedding method to predict potential bitter peptides. As a result of extensive testing and optimization of multiple feature extraction approaches, using three distinct algorithms, we identified UniRep + BiLSTM_106 as the best fusion feature set. Additional feature selection, using LGBM classifier input, allowed us to develop a robust model. Results of the 10-fold cross-validation and the analysis of the results obtained through independent testing showed that iBitter-DRLF can effectively predict bitter peptides in protein hydrolysates or amongst artificially synthesized peptide therapeutics. Based on independent test results, iBitter-DRLF significantly outperformed existing predictors. Finally, to facilitate the use of the algorithm by other scientists, we built an iBitter-DRLF webserver. We hope that the use of iBitter-DRLF prediction of bitter peptides can improve adherence with taking nutritional supplements and peptide therapeutics in the future and advance drug development and nutrition research.

This work used deep representation learning [39,40] features to improve the predictive performance of the model. Although the exact physicochemical relevance of these features is unclear, this does not prevent the successful use of this method for computational predictions in peptide and protein sequence analysis.

## Figures and Tables

**Table 1:** Performance metrics of three different deep representation learning features using three machine learning models.

Feature	Model	Dim	10-Fold Cross-Validation							Independent Test						
			ACC	MCC	Sn	Sp	F1	auPRC	auROC	ACC	MCC	Sn	Sp	F1	auPRC	auROC
SSA ^b^	SVM ^c^	121	0.826	0.652	0.836	0.816	0.828	0.89	0.898	**0.883 ** ^a^	**0.766**	0.891	0.875	0.884	0.951	0.944
	LGBM ^c^		0.787	0.575	0.816	0.758	0.793	0.874	0.886	0.859	0.722	**0.906**	0.812	0.866	0.949	0.941
	RF ^c^		0.791	0.584	0.828	0.754	0.798	0.848	0.865	0.82	0.644	0.875	0.766	0.83	0.934	0.922
UniRep ^b^	SVM ^c^	1900	**0.865**	**0.73**	**0.867**	**0.863**	**0.865**	0.937	**0.931**	0.867	0.735	0.844	**0.891**	0.864	0.952	0.948
	LGBM ^c^		0.84	0.68	0.828	0.852	0.838	**0.939**	0.93	0.867	0.735	0.844	**0.891**	0.864	0.953	**0.952**
	RF ^c^		0.842	0.684	0.836	0.848	0.841	0.927	0.92	0.844	0.688	0.828	0.859	0.841	0.946	0.943
BiLSTM ^b^	SVM ^c^	3605	0.818	0.637	0.82	0.816	0.819	0.91	0.912	**0.883**	**0.766**	**0.906**	0.859	**0.885**	**0.956**	0.951
	LGBM ^c^		0.855	0.711	0.863	0.848	0.857	0.924	0.926	0.836	0.673	0.812	0.859	0.832	0.95	0.95
	RF ^c^		0.818	0.637	0.828	0.809	0.82	0.9	0.908	0.844	0.688	0.844	0.844	0.844	0.954	0.949

^a^ Best performance values are in bold and are underlined. ^b^ SSA: soft symmetric alignment; UniRep: unified representation; BiLSTM: bidirectional long short-term memory. ^c^ SVM: support vector machine; LGBM: light gradient boosting machine; RF: random forest.

**Table 2:** Performance metrics of fusion features developed using three machine learning models in 10-fold cross-validation and in independent tests.

Feature	Model	Dim	10-Fold Cross-Validation							Independent Test						
			ACC	MCC	Sn	Sp	F1	auPRC	auROC	ACC	MCC	Sn	Sp	F1	auPRC	auROC
SSA + UniRep ^b^	SVM ^c^	2021	0.861	0.723	**0.875 ** ^a^	0.848	0.863	0.929	0.927	0.867	0.734	0.859	0.875	0.866	0.954	0.952
	LGBM ^c^		0.840	0.680	0.848	0.832	0.841	0.933	0.924	0.859	0.719	0.859	0.859	0.859	0.960	0.958
	RF ^c^		0.838	0.676	0.840	0.836	0.838	0.923	0.917	0.867	0.735	0.844	0.891	0.864	0.955	0.954
SSA + BiLSTM ^b^	SVM ^c^	3726	0.836	0.672	0.848	0.824	0.838	0.915	0.917	0.883	0.766	0.859	0.906	0.880	0.943	0.947
	LGBM ^c^		0.848	0.696	0.859	0.836	0.849	0.927	0.927	0.875	0.751	0.906	0.844	0.879	0.961	0.957
	RF ^c^		0.824	0.649	0.832	0.816	0.826	0.906	0.911	**0.898**	0.797	0.891	0.906	**0.898**	0.959	0.951
UniRep + BiLSTM ^b^	SVM ^c^	5505	0.844	0.688	0.859	0.828	0.846	0.921	0.926	0.891	0.783	**0.922**	0.859	0.894	0.966	0.962
	LGBM ^c^		0.863	0.727	0.871	0.855	0.864	0.932	0.935	0.870	0.737	0.859	0.886	0.887	**0.972**	0.958
	RF ^c^		0.832	0.664	0.844	0.820	0.834	0.932	0.930	0.875	0.750	0.859	0.891	0.873	0.963	0.960
SSA + UniRep + BiLSTM ^b^	SVM ^c^	5626	**0.871**	**0.742**	0.863	**0.879**	**0.870**	0.943	0.941	0.891	0.783	**0.922**	0.859	0.894	0.940	0.943
	LGBM ^c^		0.855	0.711	0.844	0.867	0.854	**0.945**	**0.942**	**0.898**	0.797	0.891	0.906	**0.898**	0.971	**0.971**
	RF ^c^		0.840	0.680	0.848	0.832	0.841	0.926	0.925	**0.898**	**0.799**	0.859	**0.937**	0.894	0.963	0.957

^a^ Values representing the best performance values are in bold and are underlined. ^b^ SSA + UniRep: SSA features combined with UniRep features; SSA + BiLSTM: SSA features combined with BiLSTM features; UniRep + BiLSTM: UniRep features combined with BiLSTM features; SSA + UniRep + BiLSTM: all the above features are combined. ^c^ SVM: support vector machine; LGBM: light gradient boosting machine; RF: random forest.

**Table 3:** Performance metrics of individual features and fused features, according to the machine learning methods used to derive them.

Feature	Model	Dim	10-Fold Cross-Validation							Independent Test						
			ACC	MCC	Sn	Sp	F1	auPRC	auROC	ACC	MCC	Sn	Sp	F1	auPRC	auROC
SSA ^b^	SVM ^c^	53	0.820	0.641	0.840	0.801	0.824	0.910	0.909	0.914	0.829	0.937	0.891	0.916	0.948	0.941
	LGBM ^c^	77	0.816	0.634	0.848	0.785	0.822	0.877	0.892	0.883	0.768	0.922	0.844	0.887	0.947	0.940
	RF ^c^	16	0.805	0.610	0.820	0.789	0.808	0.860	0.881	0.867	0.734	0.875	0.859	0.868	0.888	0.894
UniRep ^b^	SVM ^c^	65	0.875	0.750	0.875	0.875	0.875	0.946	0.943	0.906	0.813	0.891	0.922	0.905	0.952	0.952
	LGBM ^c^	313	0.854	0.707	0.855	0.852	0.854	0.946	0.938	0.914	0.829	0.891	0.937	0.912	0.954	0.948
	RF ^c^	329	0.836	0.672	0.824	0.848	0.834	0.918	0.908	0.891	0.785	0.844	0.937	0.885	0.958	0.957
BiLSTM ^b^	SVM ^c^	344	0.820	0.641	0.824	0.816	0.821	0.913	0.915	0.922	0.844	0.937	0.906	0.923	0.955	0.956
	LGBM ^c^	339	0.871	0.742	0.883	0.859	0.873	0.925	0.929	0.906	0.813	0.906	0.906	0.906	0.969	0.966
	RF ^c^	434	0.830	0.660	0.836	0.824	0.831	0.906	0.914	0.898	0.797	0.906	0.891	0.899	0.957	0.950
SSA + UniRep ^b^	SVM ^c^	62	0.865	0.730	0.863	0.867	0.865	0.944	0.942	0.914	0.828	0.906	0.922	0.913	0.958	0.957
	LGBM ^c^	106	0.881	0.762	0.887	0.875	0.882	**0.961**	0.957	0.891	0.783	0.859	0.922	0.887	0.952	0.947
	RF ^c^	47	0.838	0.676	0.859	0.816	0.841	0.937	0.931	0.906	0.816	0.859	0.953	0.902	0.956	0.947
SSA + BiLSTM ^b^	SVM ^c^	267	0.836	0.672	0.836	0.836	0.836	0.910	0.911	0.914	0.828	0.906	0.922	0.913	0.956	0.952
	LGBM ^c^	317	0.861	0.723	0.875	0.848	0.863	0.924	0.929	0.906	0.813	0.906	0.906	0.906	0.962	0.958
	RF ^c^	176	0.832	0.664	0.848	0.816	0.835	0.922	0.925	0.906	0.813	0.906	0.906	0.906	0.959	0.952
UniRep + BiLSTM ^b^	SVM ^c^	186	0.873	0.746	0.887	0.859	0.875	0.932	0.934	0.914	0.829	0.937	0.891	0.916	0.961	0.965
	LGBM ^c^	106	**0.889** ^a^	**0.777**	**0.891**	**0.887**	**0.889**	0.947	**0.952**	**0.944**	**0.889**	0.922	**0.977**	**0.952**	**0.984**	**0.977**
	RF ^c^	45	0.871	0.742	0.871	0.871	0.871	0.937	0.941	0.938	0.875	0.938	0.938	0.938	0.976	0.971
SSA + UniRep + BiLSTM ^b^	SVM ^c^	336	0.881	0.762	0.883	0.879	0.881	0.940	0.942	0.922	0.845	**0.953**	0.891	0.924	0.942	0.946
	LGBM ^c^	285	0.881	0.762	0.891	0.871	0.882	0.951	0.947	0.938	0.875	0.922	0.953	0.937	0.969	0.969
	RF ^c^	192	0.863	0.727	0.859	0.867	0.863	0.932	0.932	0.922	0.844	0.906	0.937	0.921	0.970	0.967

^a^ Best performance values are in bold and are underlined. ^b^ SSA: soft symmetric alignment; UniRep: unified representation; BiLSTM: bidirectional long short-term memory. SSA + UniRep: SSA features combined with UniRep features; SSA + BiLSTM: SSA features combined with BiLSTM features; UniRep + BiLSTM: UniRep features combined with BiLSTM features; SSA + UniRep + BiLSTM: all the above features are combined. ^c^ SVM: support vector machine; LGBM: light gradient boosting machine; RF: random forest.

**Table 4:** Comparison of performance metrics of bitterness prediction using iBitter-DRLF and alternative state-of-the-art methods while testing independent samples.

Classifier	ACC	MCC	Sn	Sp	auROC
iBitter-DRLF	**0.944 ** ^a^	**0.889**	0.922	**0.977**	**0.977**
iBitter-Fuse	0.930	0.859	**0.938**	0.922	0.933
BERT4Bitter	0.922	0.844	**0.938**	0.906	0.964
iBitter-SCM	0.844	0.688	0.844	0.844	0.904
MIMML	0.938	0.875	**0.938**	0.938	0.955

^a^ Best performance metrics are shown in bold and are underlined.

## Data Availability

The dataset for model training and testing is available at http://public.aibiochem.net/peptides/BitterP/ or https://github.com/Shoombuatong2527/Benchmark-datasets (accessed on 30 May 2022).

## Supplementary Materials

The following supporting information can be downloaded at: https://www.mdpi.com/article/10.3390/ijms23147877/s1.
