# Supplementary material for: Identify Bitter Peptides by Using Deep Representation Learning Features
Source: Int J Mol Sci. 2022 Jul 17;23(14):7877. doi: 10.3390/ijms23147877 (PMC9315524; doi:10.3390/ijms23147877)
Supplement: Supplementary file 1 [file ijms-23-07877-s001.zip › ijms-1774755-Supplemental figure.pdf]

Supplemental figures:

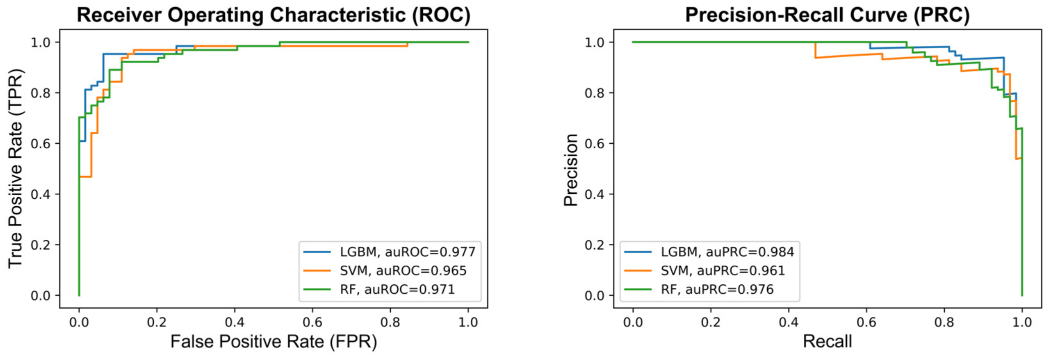

Supplemental Figure S1. The ROC curve and PRC curve of independent test of three machine learning models based on UniRep_BiLSTM best features encoding. The ROC curve and PRC curve of independent test of three machine learning models based on UniRep_BiLSTM top K feature encoding. For LGBM, SVM, RF, the K value is 106, 186 and 45 independently.

iBitter-DRLF

A novel prediction tool for bitter peptides based on deep learning representation feature methods

Home| Server| Dataset

Introduction

A bitter taste often identifies hazardous compounds and it is generally avoided by most animals and humans. Bitterness of hydrolyzed proteins is caused by the presence of bitter peptides. To improve palatability, bitter peptides need to be identified experimentally in a time-consuming and expensive process, before they can be removed or degraded. Here, we report the development of a machine learning prediction method, ***iBitter-DRLF***, which is based on a deep learning pre-trained neural network feature extraction method. It uses three sequence embedding techniques, soft symmetric alignment (SSA), unified representation (UniRep), and bidirectional long short-temp memory (BiLSTM). The results showed that the use of deep representation learning greatly improves the ability of the model to identify bitter peptides, achieving accurate prediction based on peptide sequence data alone.

Framework

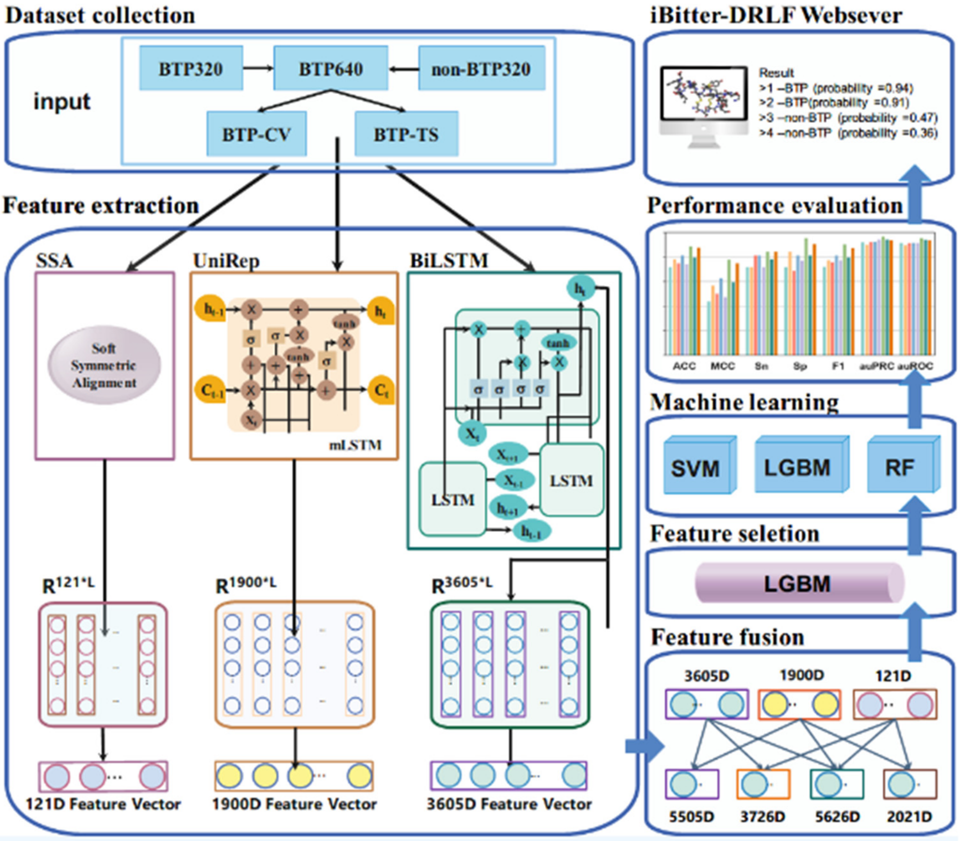

Supplemental Figure S2. Screenshots of the iBitter-DRLF webserver. Users can access the webserver by entering https://www.aibiochem.net/servers/iBitter-DRLF/, which would bring us to the webpage as shown in Figure S2.

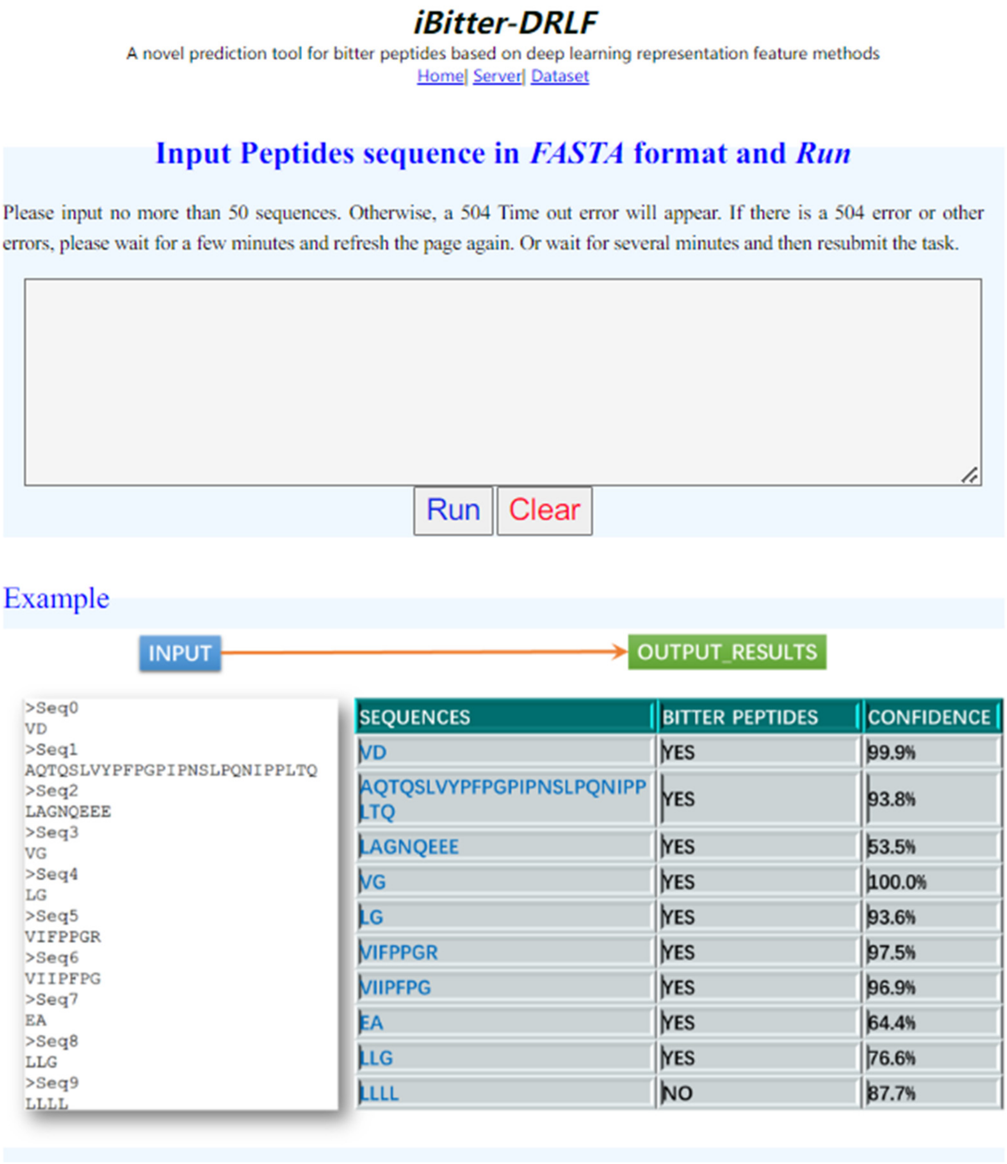

Supplemental Figure S3. Screenshots of the iBitter-DRLF webserver. Users can begin the prediction process by entering the query sequence into the text box and clicking the "Run" button; this step typically takes a few seconds for the server to process the task.

iBitter-DRLF

A novel prediction tool for bitter peptides based on deep learning representation feature methods

Home| Server| Dataset

Results

SEQUENCES	BITTER PEPTIDES	CONFIDENCE
ETWRTEAPSATGQASSLLGGRLLGQ	YES	96.27%
LGGIVSAVKKIVDFLG	YES	99.97%
GRKKRRQRRRGGWMWVTNLRTD	YES	97.48%
FAKLAKKALAKLL	YES	94.21%
KIAKVALAKLGIGAVLKVLTTGL	YES	96.78%
FLGALFKALSKLL	YES	83.27%
PLLQATLGGGS	NO	74.26%
FAKLLALALKLKL	YES	97.57%
AAVPIVNLKDELLFPSWEALFSGSE	YES	98.43%
GKWKKILGKLIR	NO	76.04%
GFFALIPKIISSPLFKTLLSAVGSALS	YES	93.10%
PAWRKARRWAWRMKKLAA	YES	80.51%

Supplemental Figure S4. Screenshots of the iBitter-DRLF webserver. After the prediction process is completed, the results are displayed on the webserver.
