# Supplementary material for: Identify Bitter Peptides by Using Deep Representation Learning Features
Source: Int J Mol Sci. 2022 Jul 17;23(14):7877. doi: 10.3390/ijms23147877 (PMC9315524; doi:10.3390/ijms23147877)
Supplement: Supplementary file 1 [file ijms-23-07877-s001.zip › ijms-1774755-Supplemental table.pdf]

Supplemental table legends:

Supplemental Table S1. Performance metrics of the UniRep+BiLSTM feature analyzed by different models using hyperparameters and default parameters. Results using selected hyperparameters invariably matched or outperformed default ones.

	Model (parameters)	Dim	ACC	MCC	Sn	Sp	F1	auPRC	auROC
10-fold CV	SVM[b] (C=1.00, g=0.01)	186	0.850	0.702	0.840	0.860	0.848	0.922	0.922
	SVM[b] (C=2.44, g=0.0035)	186	0.873	0.746	0.887	0.859	0.875	0.932	0.934
	LGBM[b] (depth=8, n_estimators=120)	106	0.871	0.746	0.863	0.879	0.870	**0.955**	**0.952**
	LGBM[b] (depth=3, n_estimators= 75)	106	**0.889**[a]	**0.777**	**0.891**	**0.887**	**0.889**	0.947	**0.952**
	RF[b] (Nleaf=1, n_estimators=120)	45	0.858	0.717	0.859	0.856	0.857	0.942	0.943
	RF[b] (Nleaf=2, n_estimators=300)	45	0.871	0.742	0.871	0.871	0.871	0.937	0.941
Independent test	SVM[b] (C=1.00, g=0.01)	186	0.906	0.813	0.906	0.906	0.906	0.963	0.965
	SVM[b] (C=2.44, g=0.0035)	186	0.914	0.829	0.937	0.891	0.916	0.961	0.965
	LGBM[b] (depth=8, n_estimators=120)	106	0.883	0.766	0.875	0.891	0.882	0.960	0.962
	LGBM[b] (depth=3, n_estimators= 75)	106	**0.944**	**0.889**	0.922	**0.977**	**0.952**	**0.984**	**0.977**
	RF[b] (Nleaf=1, n_estimators=120)	45	0.901	0.802	0.914	0.887	0.930	0.973	0.964
	RF[b] (Nleaf=2, n_estimators=300)	45	0.938	0.875	**0.938**	0.938	0.938	0.976	0.971

[a]Best performance values are in bold and are underlined.
[b]SVM: support vector machine; LGBM: light gradient boosting machine; RF: random forest.
